# Investigating Behavioral and Psychophysiological Reactions to Conflict-Related and Individualized Stimuli as Potential Correlates of Repression

**DOI:** 10.3389/fpsyg.2017.01511

**Published:** 2017-09-14

**Authors:** Henrik Kessler, Anna Christine Schmidt, Oliver Hildenbrand, Daniela Scharf, Aram Kehyayan, Nikolai Axmacher

**Affiliations:** ^1^Department of Psychosomatic Medicine and Psychotherapy, LWL-University Hospital, Ruhr-University Bochum Bochum, Germany; ^2^Department of Epileptology, University of Bonn Bonn, Germany; ^3^Department of Medical Psychology, University of Bonn Bonn, Germany; ^4^Department of Neuropsychology, Institute of Cognitive Neuroscience, Faculty of Psychology, Ruhr-University Bochum Bochum, Germany

**Keywords:** repression, individualized stimuli, free association, operationalized psychodynamic diagnosis, emotion regulation

## Abstract

**Background:** Repression is considered as a central defense mechanism in psychodynamic theory. It refers to the process by which “unbearable” mental contents (e.g., those related to internal conflicts) are kept out of consciousness. The process of repression is probably closely related to concepts of emotion regulation derived from a different theoretical background. This relationship is particularly relevant because it relates repression to current research in the affective neurosciences as well as to experimental studies on emotion regulation. Due to its complex and highly individual nature, repression has been notoriously difficult to investigate. We investigated repression with an individualized experiment in healthy subjects in order to establish methods to study repression in clinical populations. To this end we operationalized repression using individualized experimental conditions, and then studied potential behavioral [memory and reaction time (RT)] and psychophysiological correlates [skin conductance response (SCR)].

**Method:** Twenty-nine healthy female subjects were asked to freely associate to individualized cue sentences. Sentences were generated from individual psychodynamic interviews based on operationlized psychodynamic diagnosis (OPD), and were comprised of three different types: positive, negative non-conflictual, and negative conflict-related sentences. Subjects were asked to name the first three associations coming into their mind. Afterward, the remaining time was used for free association. SCR during each association trial and RT of the first given association were recorded. The memory for the first three associations was subsequently tested in an unexpected recall.

**Results:** Associations to conflict-related cue sentences were associated with longer RTs and increased SCRs. Moreover, the unexpected recall task showed memory for these associations to be reduced.

**Conclusion:** We interpret these findings as possible correlates of repression, in line with a history of experimental research into repression using non-individualized cues. Consequently, we suggest that this experimental paradigm could serve to investigate repression in clinical populations.

## Introduction

Repression is a central concept of psychodynamic theories on psychosomatic and neurotic symptoms. It refers to the mechanism by which mental contents that are related to internal conflicts are made unconscious (Freud, 1957a; Person et al., 2005; Wöller and Kruse, 2014). Repressed mental contents are less accessible to conscious processing and still underlie dynamic processes. They might, for instance, generate associated material and intrude into awareness. In the case, that repressed material gains access to consciousness, it elicits a secondary repression excluding it once more from consciousness.

Throughout our lifetimes various internal conflicts arise, e.g., conflicts of self-value, or conflicts between the desire for autarchy and the need for care. Of course, not all of these conflicts lead to pathological symptoms, but if the theme of such a conflict is touched upon in a real-life situation, it induces a plethora of reactions on the behavioral, cognitive, emotional, and somatic levels (at the very least, physiological arousal). Those reactions typically call for regulatory processes, ranging from deliberate and conscious emotion regulation (e.g., reappraisal; Gross, 2002), to less conscious defense mechanisms (e.g., repression; Brenner, 1982; Mentzos, 1984; Person et al., 2005). Repression is considered a major defense mechanism and hence an important concept guiding psychodynamic therapy (Person et al., 2005). As one of the first defense mechanisms postulated by Sigmund Freud and Josef Breuer repression was initially closely associated to “hysteria,” where symptoms (e.g., paralysis or involuntary seizure-like motor symptoms) were interpreted as a bodily converted form of oedipal unwanted feelings, thoughts or desires (Freud, 1955). This conceptualization of repression was later modified and extended by Freud, when he defined it as the process “of rejecting and keeping something out of consciousness” (Freud, 1957a). Accordingly, it was also attributed to other psychopathologies such as phobias and obsessive neuroses. Later on, repression has been further differentiated from other defense mechanisms (Freud, 1992) such as, for example, affect isolation (i.e., a subject does not experience the affective valence of a thought or a memory), displacement (i.e., a subject relocates an important issue, e.g., anger about someone, toward another person) or splitting (i.e., a subject fails to integrate two, perhaps contradicting, sides of a person). In general, the assessment of defense mechanisms is a central part of psychodynamic diagnosis, where they are additionally evaluated with regards to their flexibility and the ensuing costs of reality check. In our study, we define repression technically as a defense mechanism and thematically broadly in the original sense of “rejecting and keeping something out of consciousness” (Freud, 1957a). It is hence associated with a reduced memory for unpleasant or forbidden thoughts, affects and wishes. With regard to the specificity of our approach for investigating repression rather than other defense mechanisms, we mainly attempted to operationalize repression, but do not exclude that other defenses are induced by the autobiographical conflict sentences we presented as well. This is particularly the case because different defenses often do not occur in strict isolation, but in combination.

In line with a psychodynamic approach, very important findings concerning repression have been gained by the investigation of neurological patients suffering from anosognosia for hemiplegia (Ramachandran, 1995; Kaplan-Solms and Solms, 2000; Nardone et al., 2008; Turnbull et al., 2014). Here, the patients’ neglect of the neurological paralysis is associated to a dynamic process and hence has been interpreted as a form of defense mechanism: these patients deny their paralysis, however under certain circumstances they indeed can consciously access the fact, that for example their arm is paralyzed. This knowledge about their suffering is kept out of consciousness but can access awareness.

Importantly, cognitive approaches to the study of repression have mainly used “directed forgetting” or “think/no-think” paradigms (Anderson et al., 2004; Depue et al., 2007; Wylie et al., 2008). In these paradigms, subjects are instructed to voluntarily *suppress* unwanted memories, often tested by word pairs that are acquired in a learning phase and then either exercised (“think”-items) or suppressed (“no-think”-items). In a final recall phase the suppressed items are less likely to be remembered compared to control items (i.e., baseline items, which were learned in the first phase but not further trained afterward). This paradigm has been investigated intensely and suppression effects were frequently replicated, either for neutral, affective or autobiographical stimulus material (Depue et al., 2007; Herbert and Sütterlin, 2012; Stephens et al., 2013; Küpper et al., 2014; Noreen and MacLeod, 2014; Noreen et al., 2016). Neuroimaging studies employing this paradigm found that voluntary suppression of memory retrieval in the no-think condition was associated with increased activation of the lateral prefrontal cortex and reduced activity in the hippocampus (Anderson et al., 2004; Benoit and Anderson, 2012). This was interpreted as a down-regulation of declarative memory systems by executive control functions.

However, this important approach contrasts with the notion of repression in a clinical context, which is considered to be an unconscious, unintentional and automatic process that occurs when conflictual material arises and is hence closely related to conflict detection (Shevrin, 1990; Freud, 1992; Jones, 1993; Axmacher et al., 2010; Wöller and Kruse, 2014).

Historically, the scientific study of repression in a clinical context started with Jung’s (1918) experiments. Jung assumed that the physiological arousal and hesitation that accompanied subjects’ free association to certain cue words indicated that those words were related to internal conflicts, and that the contents triggered by those words were automatically repressed. His findings are consistent with recent studies of repression and internal conflicts, which have been found to be associated with autonomic arousal (Person et al., 2005). Also in this vein, Levinger and Clark (1961), Rossmann (1984), and Köhler and Wilke (1999) found impaired declarative memory for free associations that were accompanied by high levels of autonomic arousal.

The central idea in these studies is that the technique of free association reduces censorship (Freud, 1957b), which allows previously repressed memory contents, besides their above mentioned dynamic properties, to intrude into consciousness, i.e., to “emerge.” These memories then lead to increased autonomic arousal and the secondary repression of those contents (i.e., the repressed memory contents and their associations), which become less accessible for subsequent recall. Indeed, Köhler and Wilke (1999) noted that a limitation of these studies was that effects were only analyzed on a group level, which excludes ideas about the subjective impact of the repressed material. The authors addressed this issue in their work by analyzing intraindividual associations among the three dependent variables. However, another approach, which we wanted to address with this study, was to investigate subjectively meaningful and individually tailored material for each of the subjects.

Expanding on these early findings, we investigated the behavioral, physiological, and neural correlates of free associations to cue words and to conflict-related cue sentences in two functional magnetic resonance imaging (fMRI) studies (Schmeing et al., 2013). In both studies, high autonomic arousal [measured via increased skin conductance responses (SCRs)] and delayed responses [longer reaction times (RTs)] during free association predicted forgetting of these associations in a subsequent recall task, confirming previous findings. Interestingly, associations to conflict-related sentences were accompanied by increased activation of the anterior cingulate cortex (ACC) and deactivation of the hippocampus (HC). Strengthening our findings, a re-analysis revealed that autonomic arousal and ACC activity were more pronounced in those subjects in whom the conflict sentences touched upon an autobiographically relevant internal conflict (Kehyayan et al., 2013).

The main limitation of these previous studies, though, is the unspecific nature of the cue sentences. Although derived from psychodynamic theory and reflecting typical internal conflicts, sentences were fairly general and were the same for all subjects. When investigating highly subjective and complex phenomena, like potential internal conflicts and the repression thereof, the experimental design should be individualized and adequately consider subjects’ idiosyncratic experiences and reactions (Shevrin et al., 1992; Kessler et al., 2011, 2012, 2013).

The study reported here continues this line of research using an individualized experimental paradigm. Twenty-nine healthy female subjects were interviewed by an expert clinician according to the system of operationalized psychodynamic diagnosis (OPD; OPD-Task-Force, 2008) yielding personally relevant life events and individual conflict themes. This complex material was then used to derive stimulus sentences to be presented in the MRI scanner. Stimulus sentences referred either to (1) positive life events, (2) negative and distressing life events, unrelated to psychodynamic conflicts, or (3) conflict-related negative life events. We hypothesized that free associations to sentences describing conflict-related themes, i.e., individual “sore spots,” call for regulatory processes such as repression and should therefore be accompanied by enhanced physiological arousal (higher SCR), resistance (longer RT), and impaired memory for the association in an unexpected subsequent recall. Additionally, we suspected that not only the free associations themselves, but also contextual (background) cues presented together with the conflict sentences, would be remembered less well.

## Materials and Methods

The study was approved by the local medical ethics committee (“Ethikkommission an der Medizinischen Fakultaet der Rheinischen Friedrich-Wilhelms-Universitaet Bonn”), according to the latest version of the Declaration of Helsinki, and all subjects provided written informed consent.

### Participants

Participants were recruited through notifications on the homepage of University of Bonn Students’ Service. The return rate of interest was highly skewed toward a higher proportion of females, and thus we decided to only invite female participants. They were paid 45€ for the interview and the collection of emotionally negative and positive life-events, and 10€ per hour for the experiment (total time of the experiment 3.5–4 h). Participants were right-handed, native German speakers with normal, or corrected-to-normal vision, and without current or past neurological or psychiatric diseases according to self-reported medical history. In addition, acute depressive symptomatology was assessed using the Beck-Depression-Inventory (Hautzinger et al., 2006) and a BDI sum score above the conventional clinical cutoff for evidence of at least mild depression (BDI > 19) was defined as an exclusion criterion for the study. However, no participant had a BDI value above this cut-off.

We tested 29 subjects (age: *M* = 24.2, *SD* = 3.1). The experiment took place in the functional MRI scanner. However, functional MRI data are not reported here. Three subjects were excluded from SCR data analysis due to technical problems. Thus, SCR results are based on 26 participants.

### OPD Interview and Stimulus Generation

Participants were asked to write down six emotionally negative and six emotionally positive life events that they could recollect very vividly and that still elicited strong emotions. Then they mailed their document to the interviewer, a trained psychodynamic psychotherapist (DS). The OPD interview was based on the information about these life events. The system of OPD comprises a semi-structured interview based on five axes: axis I (experience of illness and prerequisites for treatment), axis II (interpersonal relations), axis III (psychodynamic conflicts), axis IV (psychological structure), and axis V (syndromal diagnosis according to ICD-10). In our study, axes II and III were most relevant for the generation of individualized cue sentences. OPD is an open psychodynamic interview in nature but provides flexible guidelines to ensure that the relevant information is obtained. Details can be found in the recent OPD manual (OPD-Task-Force, 2008). The interviewer distinguished conflict-related life events from non-conflict related negative and positive life events, and assigned the conflict-related life events to one or more conflict dimensions of the OPD conflict scale (see **Table 1**). Such conflicts would be expected even in our sample of healthy participants, because the existence of a conflict theme does not necessarily lead to clinically relevant symptoms. More often a conflict leads to sub-clinical tensions and distress, or it becomes (fairly) integrated into one’s everyday life.

**Table 1:** Overview of OPD conflicts (derived from OPD-Task-Force, 2008).

		Brief description
C1	Individuation vs. dependency	Existential importance of attachment and relationship. Relationship is oscillating between extremes of yearning for close relationship and symbiotic closeness (dependency), and striving for explicit independence and marked distance (individuation). (…) Seeking of closeness and attachment at all cost, versus exaggerated independence and forced avoidance of attachments.
C2	Submission vs. control	The central motive is to dominate the other, or to submit to the other. Open or latent aggressive impulses play a central role. Submission and control are non-adaptive extremes on the continuum of being able “to be guided,” or “to guide others,” respectively. Behavior norms, and other personal and societal rules are given a high value.
C3	Desire for care vs. autarchy	Desire for care versus autarchy refers to the fundamental need of individuals to obtain something, to be assured of attention and care, or to give attention and care, as opposed to not needing any care. (…) Loosing something or someone plays a central role as a trigger situation.
C4	Conflicts of self-value	Self-worth versus object worth as the extreme poles of the theme “being able to question oneself,” and “to attach a value to oneself.” Subjects consider themselves constantly inferior or superior in regard to others and can’t find the right balance between those extremes. The conflict may show as a trait (“narcissistic personality”).
C5	Guilt conflicts	Constant tendency to attribute blame to others or to blame oneself; excessive taking of responsibility, or shifting of guilt and responsibility onto others.
C6	Oedipal sexual conflicts	Difficulties in self-value considering specifically the role as a woman or man. The extreme poles are characterized by rivalry versus identification with gender-specific roles, wanting to be someone as a woman or man versus keeping in the background, being able to enjoy sexual pleasure versus sexual abstinence.
C7	Identity conflicts	Delineable, but contradictory self-representations (“identities”), chronic struggle for identity and well-being, concealment of identity dissonance.

Next, the interviewer wrote down case vignettes and sent them to a second rater (ACS or OH), who then created stimulus sentences: six positive, six negative and six conflict-related sentences, summing up to a total of 18. These sentences were discussed in a rating group, consisting of the interviewer (DS), the person who generated the sentences (ACS or OH), and another psychotherapist and experienced OPD trainer (HK).

We did not include “neutral” sentences in the study design because we wanted to contrast sentences with a similar emotional and cognitive load.

Sentences were standardized and did not differ between categories concerning sentence length and readability (“Lesbarkeitsindex,” LIX; Björnsson, 1968; see **Table 2**).

**Table 2:** Mean scores (M) and standard deviations (SD) and statistics of word count and readability index of each sentence type.

	Conflict sentence	Negative sentence	Positive sentence			
	*M (SD)*	*M (SD)*	*M (SD)*	*F*	*df*	*p*
Word count	18.35 (0.955)	18.18 (0.920)	18.13 (1.07)	0.910	2,27	0.414
Readability index	39.56 (2.89)	39.55 (2.57)	39.49 (2.74)	0.084	2,27	0.919

*Readability index < 40: children’s and youth literature.*

### Experimental Paradigm

**FIGURE 1:**
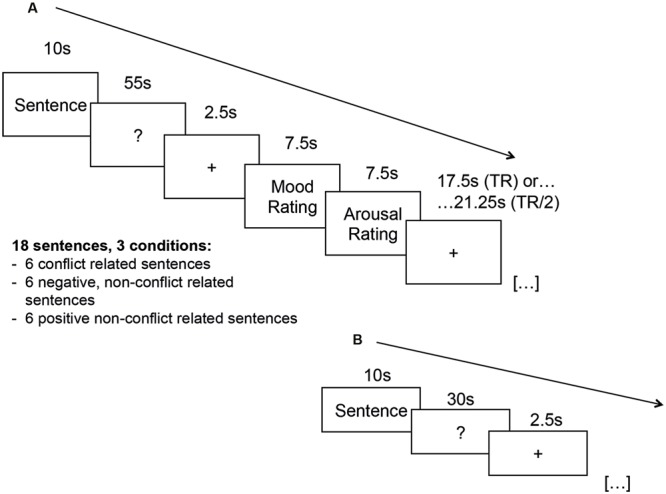
Design of **(A)** free association phase and **(B)** memory recall phase.

The experiment (see **Figure 1**) consisted of three parts: an association phase (see **Figure 1A**), a break/distraction phase, and a memory recall phase (see **Figure 1B**). Beforehand two electrodes for SCR recording were attached to the subjects’ left palm (thenar and hypothenar), and an MRI-compatible microphone was positioned in front of the lips for audio recording (Fibersound^®^ System). The paradigm was presented via MRI-compatible video goggles (Nordic Neuro Lab, Bergen, Norway).

Inside the scanner, participants had a short practice session to become acquainted with the association paradigm using standardized neutral sentences (Schmeing et al., 2013).

(1) Association phase: In order to avoid ceiling effects during subsequent memory recall, given the relatively low trial number (18 sentences), subjects were instructed to name the first *three* words which came into their minds after the presentation of each cue (this was a similar procedure to Schmeing et al., 2013). Thereafter, each trial continued for 55 s following the onset of each sentence with a free association phase. During this period of time, participants were asked to freely speak about anything that came into their minds. They were ensured that the experimenter would not listen to these associations and that their content would only be evaluated offline in a pseudonymized manner. Additionally, each sentence was presented with a background image (5.93 cm × 2.54 cm) depicting different textures (wood, stone, rock, etc.). The allocation of pictures to sentences was randomized for each participant. This was done to test the hypothesis that implicit memory for a standardized item associated with each sentence is influenced by sentence category.

After each association trial, participants were asked to rate their current mood (“How do you feel now?”), on a scale ranging from 1 (very bad) to 5 (neutral) to 9 (very good), and their arousal (“How aroused or relaxed are you?”), on a scale ranging from 1 (relaxed) to 5 (moderately aroused) to 9 (very aroused). Then they had a break of 17.5 or 21.25 s counterbalanced among all conditions (so that the next trial started either a full TR later or half a TR later).

(2) Break (1 h) for completion of questionnaires: First, subjects completed the German version of the defense style questionnaire 40 (Schauenburg et al., 2007) which assesses on a nine-point Likert scale how much subjects agree to various statements about coping strategies. The data can then be grouped and attributed to maladaptive, adaptive, or neurotic defense mechanisms. Second, they completed the Anxiety-Coping-Inventory (Krohne and Egloff, 1999), which assesses the repressor/sensitizer-profile of the participant for the dimensions “cognitive vigilance” versus “cognitive avoidance,” for ego-/self-esteem threatening scenarios (ACI-E, four scenarios) and physically threatening scenarios (ACI-P, four scenarios). Participants were presented 10 different behavioral responses (e.g., “I try not to think about the exam” as cognitive avoidance, “I think about all possible negative consequences to be best prepared” as cognitive vigilance) and were asked to choose “yes” or “no” for each item.

Finally, subjects completed the Social Desirability Scale 17 (Stöber, 1999) to control for effects of socially desirable responsiveness on our dependent variables (i.e., RT, memory performance, SCR). The Social Desirability Scale 17 assesses on 17 items behavior patterns, which (a) are socially desirable but have a low probability to occur (e.g., “In a dispute I always stay objective and factual.”) and (b) which are socially undesirable but have a high probability to occur (e.g., “Occasionally I gossip about people behind their backs.”). Participants are instructed to agree or disagree to these statements.

(3) Memory recall: After the break, participants were given written instructions for an unexpected recall of their previous free associations. Again, they were connected to the SCR amplifier in the MRI scanner. All 18 sentences were presented in random order for 10 s. This was followed by a period of 30 s duration during which participants tried to recall the three associated words that were given in the association phase. Again, the response was recorded digitally. Before the presentation of each new stimulus, there was an inter-stimulus interval of a duration randomized between 1.5 and 3 s, during which a fixation cross was presented. Subjects were allowed to correct themselves, and were encouraged to make a guess if they were not sure about their answer. Subjects did not receive feedback whether their recall was correct.

After memory recall of the freely associated words, participants were asked to complete a recognition task for the background pictures. Each picture was paired with a similar but new picture. Participants were instructed to decide which picture had been previously presented. Timing was self-paced.

Finally, participants were presented all sentences again and were asked to indicate on Likert scales, ranging each from 1 to 5, (1) the emotional valence of each sentence; (2) how much they were affected by the sentence; (3) whether they had tried to control their feelings during reading and during the free association phase; (4) how vividly they could remember the situation which was described by the sentence; and (5) how often per week after the interview they had thought about the presented memory.

### Analysis of Reaction Time

Recording of audio files started with sentence presentation and stopped at the end of the association period (65 s). Based on the visualized audio traces (“Audacity,” version 2.0.5^1^), the onset of the first word generation was detected manually in milliseconds.

### Skin Conductance Acquisition and Analysis

Skin conductance (SC) data were collected using the default workspace of the BrainVision Recorder. Sampling rate was set to 5000 Hz. Due to MRI artifacts caused by the magnetic field and the MRI pulse generation, SC data were preprocessed with the MRI artifact correction of BrainVision Analyzer 2.0. During artifact correction, data were down-sampled to 25 Hz and low-pass filtered to 5 Hz. We extracted mean phasic SC activity during the complete association period (involving the period with the three words and the free association period; 65 s in total).

### Statistical Analyses

We tested our hypothesis with repeated measures analyses of variances (ANOVA) with sentence condition as within-subjects-factor. Planned comparisons were conducted with *t*-tests. Associations between variables were tested by Spearman correlations. All tests were two-tailed.

## Results

### Life Events, Conflicts, and Free Associations

The OPD interview allowed us to identify typical psychodynamic conflicts. **Table 3** gives an overview of the frequency of typical conflict categories (according to OPD), and the number of conflicts per subject. For 19 subjects, 2 conflict themes were found, and for 1 subject, 3 conflict themes were identified. In total, 6 of 7 OPD conflict themes occurred at least once in our sample. We did not find any participants with an identity conflict. Though these conflict themes might appear to some psychoanalytically trained readers more related to early personality than neurotic disorders, it should be noted, that the OPD conflict categories are principally detached from deficits on a structural level. Thus, every conflict theme can be associated with a neurotic or a more disintegrated personality structure. Additionally, in contrast to some views in psychoanalytic theory, repression is defined broadly in our study and hence not only related to neurotic oedipal conflicts.

**Table 3:** Frequency of conflict themes (rows) and number of conflict themes per subject (columns).

		(1) Conflict	(2) Conflict	(3) Conflict	*n* each conflict
C1	Dependence vs. autonomy	4	6	0	10
C2	Submission vs. control	7	2	0	9
C3	Desire for care vs. autarchy	9	2	0	11
C4	Conflicts of self-value	8	7	1	16
C5	Guilt conflicts	0	2	0	2
C6	Oedipal sexual conflicts	1	0	0	1
C7	Identity conflicts	0	0	0	0
		*n* total	*n* total	*n* total	
		29	19	1	

In the following section we would like to give a brief impression of the experimental stimuli by depicting negative life events, stimulus sentences, and free associations from three subjects.

### Participant 1

Participant 1 (22 years) reported autobiographical material that could be interpreted as a conflict of self-value (“C4” according to OPD). For one negative life event she described how perfect her sister (who is now at university) was at school, and that she could not understand, that their parents are proud of both of the siblings. Thus, one of her conflict sentences was as follows: “Sometimes I am surprised that my parents are equally as proud of me as they are of my sister.” The three following associations were: “Grief, childhood, problems.” The participant then continued with the following free association: “My little sister is a perfect person. She is popular, she is intelligent, she manages everything she puts her hands on. This is not how I am. I am not able to manage everything I put my hands on. And yes, these are childhood memories that are not very pleasant… I do not know why I cannot achieve what my little sister is able to achieve [*sounds as if she is crying*].” As another negative autobiographical event this participant reported, that she was very afraid and in panic of the deep water, when she had to snorkel surrounded by manta rays. Since this event is not related to her conflict theme, it was used as a control stimulus, which has a highly arousing and emotionally negative impact, but is not related to her psychodynamic conflict. Thus, one of her negative non-conflict related sentences was as follows: “In the beginning snorkeling with manta rays is very disturbing and overcharging, because I am afraid of the deep water.” The three following associations were: “Disturbing, despair, chaos.” She continued with the following free association: “Yes, we were on Fidschi and they yelled at me “quickly, get into the water” and I am afraid of deep water, so I jumped into the water and did not know where top and bottom is, and I did not know, how to regulate my breath and it was a huge mess. These rays, which you know of, that one person was impaled by one, this Australian animal-film-maker, and at the same time you also know “hey, I am on Fidschi and snorkeling with a manta ray” and it was incredibly cool and in the end I can say “hey, I overcame my fear of deep water when I snorkeled with a manta ray” and who else can say that and meanwhile I can control it better.”

### Participant 2

This participant (25 years) reported autobiographical themes and events that could be interpreted as a conflict of submission versus control (“C2” conflict according to OPD; OPD-Task-Force, 2008). One life event she reported in the interview referred to a group work project she did as part of her studies. Here, she complained about her fellow students lacking competency and her will to present a good paper. Consequently, she reported that she felt forced to correct another student’s work without informing her that she had done so. Without these corrections, she admitted that she would have felt too embarrassed to hand in the group work. We generated the following conflict sentence based on this life event: “I secretly hand in our group work results even though I do not know what my fellow student thinks about it.” The three following associations were: “Obstinacy, workaholic, perfectionism.” The participant then continued with the following free association: “Yes, in principal I am someone who works alone. However, that is linked to the fact that I only get idiots who work with me in a group. And as I am prone to be a little perfectionistic, even though I am one of the laziest people on the planet, I always want, yes, to hand in something that is as perfect as possible. This then leads to me doing it mostly on my own. I do have certain different requirements and standards compared to my classmates. And I would feel very uncomfortable handing in something in my name that I do not back fully. And yes, thus I mostly do things on my own and pretend as though the work was done by us both or all of us.” As another negative non-conflict related event she described an incident where her beloved horse suffered from a colic and had to be put down by the veterinary. Although being very negative and with high personal impact, this event had no connections to her psychodynamic conflict and was hence used in the control condition. The stimulus sentence was as follows: “After a sudden severe colic my horse unfortunately had to be put down, which makes me very sad.” The three following associations were: “Grief, missing, not being here.” She continued with the following free association: “This [event] is still following me, I think of it everyday and I miss it [the horse] every second, though we meanwhile have two new horses, they cannot replace it at all. I think, I am missing this life period as well, it [the horse] accompanied me for 10 years, my entire childhood and youth, and it not being here anymore, hurts my soul every second, because we were incredibly good friends and were very close, even if outsiders probably cannot understand that at all. But it was my best friend und we went through a lot together.”

### Participant 3

This participant (24 years) reported autobiographical material that could again be interpreted as a conflict of submission versus control (C2). Several negative life events she described were about her relationship with her boyfriend and both trying to find acceptable compromises about spending their leisure time. The subject reported that she wanted to be asked about every planned activity for a weekend because her boyfriend would be unorganized. As compensation she would allow him to decide what to cook or which film to watch. Based on this information we generated the following conflict sentence: “I always want to be in control in my relationship as I feel my boyfriend is not organized enough.” The following three associations were: “[name of boyfriend], dancing, weekend.” The participant continued with the following free association: “Yes, I only see my boyfriend on the weekends. He is a little bit disorganized, he lives for the moment; I am a planner. I like to pre-discuss what I do. And it is often like, “well, what we don’t do today, we’ll do tomorrow.” That is very annoying, but in general we find a consensus. I do not like to be stressed over the weekend just because we don’t know what we are going to do. That’s because I only get free time over the weekends. During the week I am at university all of the time, I learn a lot and then get home very late. Then I am happy if I can have some peace. If I then hear “We’re about to do ABCD all day long,” it annoys me. If I didn’t know about it beforehand then I feel stressed, and I feel stressed very quickly. I get that feeling quite often. Not only at weekends, simply because I’m not spontaneous, because I planned something else instead or am prepared for something else.” As another negative non-conflict related life event she described her daily stress when she has to travel between two distant cities, B. and M. Again, this event is arousing and emotionally negative but does not touch upon her psychodynamic conflict. The stimulus sentence was as follows: “I find traveling with bus and train from B. to M. mostly very strenuous.” The following three associations were: “Boyfriend, family, Deutsche Bahn.” She continued with the following free association: “Yes, I always have to travel from B. to M., when I visit my family or my boyfriend. Yes, Deutsche Bahn is lovely, you are constantly delayed, although you get from B. to C., you don’t catch the connecting train or the connecting train is delayed. Or on the way back, that is wonderful, you arrive, and definitely miss the connecting train, and you arrive always during this time period, when no other train is heading to B. or vice versa, it is always like that. And then Deutsche Bahn is not able to say: “Hey, could the connecting train please wait?” Yes and additionally it is a lot of traveling, almost 2 h, when I take the ICE it is one and a half, each way, that is very time consuming. I cannot learn in the train, maybe I read a bit, but even this is somehow strenuous.”

### Behavioral and Psychophysiological Data

**FIGURE 2:**
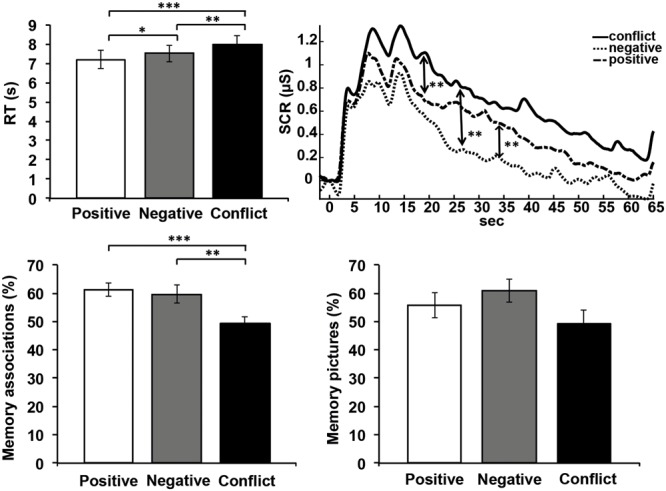
Results of behavioral and psychophysiological data (error bars depict SEM). ^∗^*p* < 0.05; ^∗∗^*p* < 0.01; ^∗∗∗^*p* < 0.001.

Descriptive statistics of behavioral and psychophysiological data are presented in **Table 4** and **Figure 2**.

**Table 4:** Mean scores (M) and standard deviations (SD) of behavioral and psychophysiological data.

		*M*	*SD*
RT (s)	Conflict	7.96	2.52
*n* = 29	Negative	7.51	2.39
	Positive	7.20	2.54
SCR (μS)	Conflict	0.648	0.138
*n* = 26	Negative	0.248	0.204
	Positive	0.447	0.255
Memory of free associations (%)	Conflict	49.18	14.16
*n* = 29	Negative	59.66	16.83
	Positive	61.30	21.81
Memory of pictures (%)	Conflict	49.43	25.39
*n* = 29	Negative	60.92	21.95
	Positive	55.75	23.69

Repeated-measures ANOVAs revealed a main effect of “condition” for RTs (*F*_2,27_ = 13.99, *p* < 0.001, $\eta_{p}^{2}$ = 0.333), SCRs (*F*_2,24_ = 35.42, *p* < 0.001, $\eta_{p}^{2}$ = 0.747) and memory for associations (*F*_2,27_ = 9.50, *p* < 0.001, $\eta_{p}^{2}$ = 0.253). No main effect was found for the memory effects for background pictures (*F*_2,27_ = 2.31, *p* = 0.120 $\eta_{p}^{2}$ = 0.146). Planned comparisons showed that RTs for the conflict condition were significantly longer than those in the negative (*t*_28_ = 3.00, *p* = 0.006) or positive conditions (*t*_28_= 4.86, *p* < 0.001), and also differed between negative and positive sentences (*t*_28_ = 2.47, *p* = 0.020). The mean phasic SCR was higher in the conflict condition than in the negative (*t*_25_ = 8.26, *p* < 0.001) or positive condition (*t*_25_ = 4.37, *p* < 0.001). Interestingly, the mean phasic SCR was lower in the negative condition than in the positive condition (see **Figure 2** and **Table 4**, *t*_25_ = -5.71, *p* < 0.001). Associations in the conflict condition were less likely to be remembered than either in the negative (*t*_28_ = -2.95, *p* = 0.006) or positive conditions (*t*_28_= -4.42, *p* < 0.001), but did not differ between the negative and positive condition (*t*_28_ = -0.614, *p* = 0.540). Repeated analyses with non-parametrical tests (Friedman and Wilcoxon) confirmed the parametrical results.

Though analysis of variances did not show a main effect of condition on memory for background pictures, the mean values were ordered in the expected direction (see **Figure 2**).

Subjective ratings of valence, mood, arousal, affection, and emotion regulation differed between conflict-related, negative, and positive sentences (see **Table 5**; all *F*_2,27_ > 9, all *p* < 0.002). Importantly, however, no significant differences in ratings were found between conflict-related sentences and negative sentences (all *t*_28_ < 1.80, all *p* > 0.070), indicating that they were evaluated as similarly negative on a conscious level. Moreover, subjective ratings of vividness (see **Table 5**) and recollection frequency of the events since interview described in the sentences differed among the three sentence types (vividness: *F*_2,27_ = 24.91, *p* < 0.001, $\eta_{p}^{2}$ = 0.649; recollection: *F*_2,23_ = 7.74, *p* = 0.003, $\eta_{p}^{2}$ = 0.402). Conflict-related and negative sentences were equally vividly experienced (*t*_28_ = 1.77, *p* = 0.087) and less vividly compared to positive sentences (conflict vs. positive: *t*_28_ = -4.91, *p* < 0.001; negative vs. positive: *t*_28_ = -5.95, *p* < 0.001). Interestingly, events described in conflict-related and positive sentences were equally frequently recollected since the interview (*t*_24_ = 0.047, *p* = 0.963) compared to negative sentences, that were less frequently recollected (conflict vs. negative: *t*_24_ = 3.03, *p* = 0.006; positive vs. negative: *t*_24_ = 3.85, *p* = 0.001). Additionally, we found no association between scores on the Social Desirability Scale-17 and measures of the dependent variables (i.e., RT, memory performance, and SCR for each condition) controlling for multiple comparisons (*r* < 0.459, *p* > 0.023, alpha_corrected_ = 0.006).

**Table 5:** Mean scores (M) and standard deviations (SD) of mood and arousal rating and post-experimental questionnaire.

		Conflict		Negative		Positive	
		*M*	*SD*	*M*	*SD*	*M*	*SD*
During experiment	Mood	4.85	1.02	4.87	0.887	6.73	1.05
	Arousal	5.28	1.40	5.17	1.30	4.98	1.68
Post-experimental questionnaire	Valence	2.10	0.526	2.08	0.465	4.56	0.279
	Affection	3.26	0.657	3.13	0.742	3.70	0.576
	Emotion regulation	1.77	0.833	1.60	0.659	1.29	0.458
	Vividness	3.76	0.640	3.47	0.751	4.30	0.465
	Recollection	1.41	0.856	0.792	0.647	1.40	0.707

### Questionnaire Data

Supplementary Table 1 presents descriptive statistics from the personality questionnaires. We did not find any significant correlations between the experimental effects (RT, SCR and memory for conflict-related vs. negative sentences) and questionnaire variables (see Supplementary Table 1, *r* < 0.429, *p* > 0.020, alpha_corrected_ = 0.005).

## Discussion

In our study, *N* = 29 healthy female subjects were meticulously interviewed according to the OPD (OPD-Task-Force, 2008) criteria, in order to extract positive, negative and conflict-related life events, which were used to create individualized cue sentences. Conflict-related life events were related to a typical psychodynamic conflict, i.e., individual “sore-spots.” While freely associating to conflict-related sentences, subjects had longer RTs, higher SC responses and impaired memory for the associations produced in an unexpected subsequent recall when compared with positive and negative sentences. There was also some hint of reduced memory for background pictures shown together with conflict sentences.

Our work expands upon a line of studies starting with C. G. Jung’s early experiments on free associations to cue words (Jung, 1918), which were later replicated by other teams (Levinger and Clark, 1961; Rossmann, 1984; Köhler and Wilke, 1999), as well as in our own previous works (Kehyayan et al., 2013; Schmeing et al., 2013). The study presented here further developed this paradigm by using individualized stimuli derived from an OPD interview. Overall, generating individualized stimuli was very time-consuming and closely matched to a clinical context. The reason for this effort lay in the overarching goal of our venture: we attempted to operationalize a clinically meaningful concept – repression as a major defense mechanism according to psychodynamic theory – under experimental conditions with healthy subjects, in order to make it assessable in clinical populations in the future. Psychosomatic or neurotic patients, whose disorders could be connected to psychodynamic conflicts, may benefit from the detection of such conflicts via an experimental procedure similar to the one applied in this study. The derivation of individualized stimuli from a psychodynamic clinical interview was seen as a necessary intermediate step to the eventual realization of this goal, and therefore worth the added effort.

As in previous studies (Kehyayan et al., 2013; Schmeing et al., 2013), prolonged RTs and higher SCR were considered to be indirect markers of the ongoing resistance and autonomic arousal that is elicited by conflict-related stimuli, which lead to repression. Our observations in the present study are consistent with our previous findings that used standardized conflict-related sentences. Thus, the present study expands upon these findings by showing the same effects when using individually tailored stimuli, which arguably target an individual’s conflict themes more specifically.

Previous research on behavioral responses to highly arousing negative and positive stimuli has been heterogeneous. While some studies show that arousal facilitates stimulus processing irrespective of valence (Schacht and Sommer, 2009), others suggest that emotionally negative (Zinchenko et al., 2015) or positive (Herbert et al., 2009) stimuli are processed preferentially. Unexpectedly, we also obsereved increased SC responses for positive as compared to negative sentences (see **Figure 2**). While we do not have a clear explanation for this effect, it may be (in line with the above mentioned heterogenous findings) due to the high perceived salience of the positive events, even though these events evoked smaller SC respones than the conflict-related experiences. Besides this, we introduce a further dimension of pivotal ecological relevance: the relatedness of negative stimuli to autobiographical conflicts. The results of the present study suggest that particularly ego-threatening and self-relevant stimuli (for example, those that elicit shame or guilt) are accompanied by longer RTs, which is consistent with previous studies on the RT effects of psychodynamic conflicts (Shevrin, 1990).

Importantly, subjective ratings of emotional valence and arousal did not differ between conflict-related and negative sentences. This may indicate that the differences in *autonomic* arousal (and RTs) were not paralleled by differences in *conscious* processing of valence and/or arousal, but might rather be considered correlates of unconscious or preconscious processing of valence and arousal. In this vein, we investigated two other possible confounds, the vividness and recollection of sentences. Although positive sentences were rated to be more vivid, there was no difference in vividness between negative and conflict-related sentences. The differences in memory performance between negative and conflict-related sentences can thus not simply be explained by vividness. Interestingly, positive and conflict-related sentences were equally frequently recollected since the interview, but conflict-related sentences were still remembered worse in the recall experimental session. Hence, differences in experimental (i.e., relative short-term) memory performance might not be attributed to mere effects of remembering sentences *per se* over time.

We found no association between experimental effects and results from personality questionnaires, especially not for a measure of repressive coping style. Again, this appears to indicate that the conscious self-appraisal of being more or less vigilant toward unpleasant stimuli, or of suppressing thoughts about unpleasant stimuli, does not correlate with the concept of repression, as it is operationalized in, e.g., Jung’s (1918) early experiments or in recent clinical concepts (Person et al., 2005).

Concerning the limitations of this study, the main issue is expressed in the antagonism between a naturalistic versus laboratory perspective: is the design naturalistic enough? Is it possible to capture complex psychodynamic processes like the reaction to conflict-related material and the defense mechanism of repression with any experimental (i.e., controlled) design?

On the other hand, is the design controlled enough? Subjects’ associations, and their respective memory performances, are not under the experimenter’s control. The associations themselves are – naturally – highly variable, and hence pose difficulties for the experimental design and analytic approach.

Furthermore, as mentioned in the introduction, other studies on repression also considered the *return of the repressed* by showing that neurological patients with anosognosia can overcome their unawareness of the paralysis (Ramachandran, 1995; Nardone et al., 2008; Turnbull et al., 2014). In these studies, anosognosia was not interpreted as a mere cognitive deficit in attention, but rather as resulting from defense against unbearably painful affects associated with paralysis. Psychodynamic interventions based on this reconceptualization of anosognosia were able to relieve symptoms in several patients (Kaplan-Solms and Solms, 2000), and there was some evidence that patients were implicitly aware of their deficits (Fotopoulou et al., 2010). Moreover, anosognosia has been conceptually linked to impairments of a brain system for emotion regulation (Turnbull et al., 2014), inducing regression to the more premature, and dysfunctional, process of repression.

According to the cognitive approach to repression, “suppression” (Anderson and Green, 2001), a comparative study is needed for a more differentiated conceptualization of repression. Here, the differences (e.g., return of the target material) and similarities (e.g., form of emotion regulation via memory control) of these two mechanisms warrant further analysis to improve our understanding of these forms of emotion- and self-regulation. Additionally, as different sorts of defense mechanisms tend to overlap, further research is needed to compare our operationalization of repression to other closely related defense mechanisms like denial. Importantly, in our OPD assessment, we focused on neurotic conflicts rather than structural deficits (i.e., those related to narcissistic or borderline states) because we were primarily interested in repression rather than dissociative phenomena. We cannot exclude, though, that some of the subjects we included also suffered from structural deficits, even though we screened them for current psychiatric diseases. We believe that future studies directly comparing repression and dissociative phenomena will be important.

Finally, it should be noted that our indirect markers for resistance and repression can by no means be considered exclusive indicators of such complex processes. In this study we focused on resistance and an involuntarily induced memory inhibition induced by individualized conflict-related material for a working definition of repression. In future studies, the inclusion of more physiological and neurophysiological measures (i.e., heart rate, electromyography, EEG), which are not influenced by conscious processes, could strengthen the idea that we actually capture unconscious or preconscious processes. These findings should also be independently replicated and investigated in a clinical population, which, if successful, would achieve our intended goal for a diagnostic application of this experimental design.

## Informed Consent

Written informed consent was obtained from all individual participants included in the study.

## Author Contributions

HK, ACS, OH, DS, AK, and NA conceived and designed the study. DS conducted the OPD interviews. HK, ACS, OH, and DS generated and evaluated experimental stimuli. ACS and OH performed the experiment. HK, ACS, OH, and AK analyzed the data. HK, ACS and NA drafted the paper. OH, DS, and AK revised the manuscript critically for important intellectual content.

## Conflict of Interest Statement

The authors declare that the research was conducted in the absence of any commercial or financial relationships that could be construed as a potential conflict of interest. The reviewer DM declared a past co-authorship with one of the authors, NA, to the handling Editor.

## Footnotes

1 http://audacity.sourceforge.net/
