# Supplementary material for: Investigating Behavioral and Psychophysiological Reactions to Conflict-Related and Individualized Stimuli as Potential Correlates of Repression
Source: Front Psychol. 2017 Sep 14;8:1511. doi: 10.3389/fpsyg.2017.01511 (PMC5603662; doi:10.3389/fpsyg.2017.01511)
Supplement: Supplementary file 1 [file Table_1.docx]

**Supplementary Table**

Supplementary Table 1. Mean scores (M) and standard deviations (SD) for personality questionnaire data and correlation coefficients (r) for association between questionnaire data and effects of the free association paradigm.

|  | **M** | **SD** | **r_RT effect_** | **r_SCR effect_** | **r_Memory effect_** |
| --- | --- | --- | --- | --- | --- |
| **BDI** | 7.38 | 5.05 | -0.106 | 0.111 | 0.246 |
| **DSQ mature** | 4.83 | 1.00 | -0.063 | -0.313 | -0.073 |
| **DSQ neurotic** | 4.61 | 0.999 | -0.038 | -0.130 | 0.103 |
| **DSQ immature** | 3.29 | 0.807 | 0.011 | 0.097 | 0.117 |
| **ACI-E COA** | 9.59 | 3.77 | 0.200 | 0.026 | -0.127 |
| **ACI-E VIG** | 13.45 | 4.04 | 0.235 | 0.020 | 0.070 |
| **ACI-P COA** | 11.38 | 3.12 | 0.429 | -0.006 | -0.038 |
| **ACI-P VIG** | 10.38 | 3.93 | -0.066 | 0.153 | 0.290 |

BDI = Beck’s Depression Inventory, DSQ = Defense Style Questionnaire, ACI-E = Anxiety-Coping-Inventory ego-threatening, ACI-P = Anxiety-Coping-Inventory physically threatening, COA = cognitive avoidance, VIG = cognitive vigilance; RT effect = reaction time_conflict-related_ - reaction time_negative_, SCR effect = SCR_conflict-related_ - SCR_negative_, Memory effect = memory_negative_ - memory_conflict-related_.
